# Supplementary material for: The COVID-19 Pandemic and Its Impact on Homebound Nursing Students
Source: Int J Environ Res Public Health. 2020 Oct 10;17(20):7383. doi: 10.3390/ijerph17207383 (PMC7600682; doi:10.3390/ijerph17207383)
Supplement: Supplementary file 1 [file ijerph-17-07383-s001.pdf]

Supplementary Material

Table S1. Adapted version of the Student Stress Inventory-Stress Manifestations (SSI-SM) for Nursing Students.

Items	**Not at all**	**Rarely**	**Sometimes**	**Very often**	**Completely**
1. I feel irritated					
2. Food disorders					
3. I feel afraid					
4. I feel worried					
5. I feel anxious					
6. I react defensively against others					
7. I feel burdened					
8. I have cold sweats					
9. I have itches all over my body					
10. I feel unable to face university					
11. I disregard my social relations					
12. I don’t know what to do					
13. I have a negative attitude against others					
14. I have heart palpitations					
15. I feel angry					
16. I discredit other people					
17. I have a stomachache					
18. I have difficulties interacting with others					
19. I don’t attend university because I am ill					

This version has been validated by Simonelli-Muñoz *et al.* (1) from the original version from Fimian *et al.* (2) and the Spanish adaptation by Escobar-Espejo *et al.* (3).

References

1. Simonelli-Muñoz, A.J.; Balanza, S.; Rivera-Caravaca, J.M.; Vera-Catalán, T.; Lorente, A.M.; Gallego-Gómez, J.I. Reliability and validity of the student stress inventory-stress manifestations questionnaire and its association with personal and academic factors in university students. *Nurse Educ. Today* **2018**, *64*, 156–160, doi:10.1016/j.nedt.2018.02.019.
2. Fimian, M.J.; Fastenau, P.A.; Tashner, J.H.; Cross, A.H. The measure of classroom stress and burnout among gifted and talented students. *Psychol. Sch.* **1989**, *26*, 139–53, doi:10.1002/1520-6807(198904)26:2<139::AID-PITS2310260205>3.0.CO;2-E.
3. Escobar-Espejo, M.; Blanca, M.J.; Fernandez-Baena, F.J.; Trianes Torres, M.V. Spanish adaptation of the Stress Manifestations Scale of the Student Stress Inventory (SSI-SM). *Psicothema* **2011**, *23*, 475–485.
